# Unintentional injury and its associated factors among left-behind children: a cross-sectional study

**DOI:** 10.1186/s12888-023-04964-w

**Published:** 2023-06-29

**Authors:** Zhiyu Jin, Bingsong Han, Jing He, Xinyuan Huang, Kun Chen, Jiana Wang, Zhuang Liu

**Affiliations:** 1 Department of Social Medicine, School of Health Management, China Medical University, No. 77 Puhe Road, Shenyang North New Area, Shenyang, Liaoning, People’s Republic of China; 2 Journal Center of China Medical University, China Medical University, No. 77 Puhe Road, Shenyang North New Area, Shenyang, Liaoning, People’s Republic of China

**Keywords:** Unintentional injury, Left-behind children, Cross-sectional study, China

## Abstract

**Background:**

Unintentional injuries among children and adolescents are a major public health problem worldwide. These injuries not only have negative effects on children’s physiology and psychology, but also bring huge economic losses and social burdens to families and society. Unintentional injuries are the leading cause of disability and death among Chinese adolescents, and left-behind children (LBC) are more prone to experience unintentional injury. The purpose of this study was to evaluate the type and incidence of unintentional injury among Chinese children and adolescents and explore the influences of personal and environmental factors by comparing the differences between LBC and not left-behind children (NLBC).

**Methods:**

This cross-sectional study was conducted in January and February 2019. Additionally, 2786 children and adolescents from 10 to 19 years old in Liaoning Province in China were collected in the form of self-filled questionnaires, including Unintentional Injury Investigation, Unintentional Injury Perception Questionnaire, Multidimensional Subhealth Questionnaire of Adolescent (MSQA), Negative life events, “My Class” questionnaire and Bullying/victim Questionnaire. Multiple logistic regression analysis was used to explore the factors associated with unintentional injury among children and adolescents. Binary logistic regression analysis was used to explore the factors affecting unintentional injuries between LBC and NLBC.

**Results:**

The top three unintentional injuries were falling injuries (29.7%), sprains (27.2%) and burns and scalds (20.3%) in our study population. The incidence of unintentional injuries in LBC was higher than that in NLBC. Burn and scalds, cutting injury and animal bites in LBC were higher than those in NLBC. The results show that junior high school students (odds ratio (OR) = 1.296, CI = 1.066–1.574) were more likely to report multiple unintentional injuries than primary school students. Girls (OR = 1.252, CI = 1.042–1.504) had higher odds of reporting multiple unintentional injuries. The odds of multiple injuries in children and adolescents with low levels of unintentional injury perception were higher than those in children and adolescents with high levels of unintentional injury perception (OR = 1.321, C = 1.013–1.568). Children and adolescents with a higher levels of mental health symptoms (OR = 1.442, CI = 1.193–1.744) had higher odds of reporting multiple unintentional injuries. Compared with teenagers who had never experienced negative life events, teenagers who had experienced negative life events many times (OR = 2.724, CI = 2.121–3.499) were more likely to suffer unintentional injuries many times. Low-level discipline and order (OR = 1.277, CI = 1.036–1.574) had higher odds of reporting multiple unintentional injuries. In-school adolescents who were bullied were more likely to report being injured multiple times than their counterparts who were not bullied (OR = 2.340, CI = 1.925–2.845). Low levels of unintentional injury perception, experienced negative life events and bullying had greater impacts on LBC than on NLBC.

**Conclusion:**

The survey found that the incidence of at least one unintentional injury was 64.8%. School level, sex, unintentional injury perception, subhealth, negative life events, discipline and order and bullying were associated with incidents of unintentional injury. Compared with NLBC, LBC had a higher incidence of unintentional injury, and special attention should be given to this group.

## Introduction

Unintentional injury refers to injury without purpose and consciousness, mainly including traffic injury, fall injury, burns and scalds [1]. Whether in developed or developing countries, injury has been listed as the three major diseases that seriously endanger human health and life, along with infectious diseases and chronic noncommunicable disease [2]. Unintentional injury not only causes a huge medical burden and economic losses but also brings a heavy disease burden to the family, society and country [3]. According to the global burden of disease estimates published by the World Health Organization, injuries cause 5 million deaths each year, and nearly 80% of these deaths (3.9 million) are due to unintentional injuries [4]. In addition, many people who do not die due to unintentional injury had an increased risk of lifelong disability [5]. Surveys showed that unintentional injuries account for 3 of the 15 leading causes of death among children and adolescents under 20 years of age [4]. Traffic injury, drowning, burn and scalds, falls and poisoning accounted for 60% of the causes of death in children [6]. Worldwide, the second largest cause of disabled life expectancy (YLD) of children aged 10–24 years is unintentional injury, accounting for approximately 12.0%, approximately 6.0% in high-income countries and 16.0% in other underdeveloped areas in Sub-Sahara Africa [7]. More than 95% of injury-related deaths occur in low—and middle-income countries in all age groups [5]. As one of the largest developing countries in the world, unintentional injury are the leading cause of disability and death among adolescents in China, with more than 200,000 children dying each year from unintentional injuries. Within traffic injuries, drowning and falls injuries accounted for 43.6% of the total deaths of teenagers aged 10–19 [8]. The disability-adjusted life year caused by unintentional injury in China was 10% [9]. Studies have found that if more effective prevention strategies are discussed and implemented, China can significantly reduce deaths and disability caused by unintentional injury [10].

In China, it is particularly necessary to pay attention to LBC. Since the early 1990s, the Chinese economy has entered a stage of rapid development, and uneven regional development has led to large-scale population migration. Under such a social background, parents of rural families were forced to work in other places, and the number of LBC in the whole society has increased sharply. The number of migrants and LBC at the compulsory education stage has reached 33.86 million, with close to 20.19 million LBC in rural areas [11]. At present, research on LBC in China is mainly conducted from the perspectives of pedagogy, psychology, and sociology, while research on the unintentional injuries of LBC is limited. Studies have shown that LBC are more prone to experience unintentional injury. For example, a study found that the prevalence of intentional injuries for Chinese LBC and NLBC was 34.2% and 20.8%, respectively [12]. A review of 34 cross-sectional studies found that the injury rate of LBC was 38.24%, which was higher than the 27.94% of NLBC in China [13]. In addition, an intervention study in southeast China found that LBC face a greater risk of unintentional injury [14]. Therefore, more attention should be given to the unintentional injury of LBC.

Studies have shown that the unintentional injury of children and adolescents is related to a wide range of factors that are constructed as a multilevel system. Bandura’s (1986) model of triadic reciprocal determinism considers that human behavior is not solely determined by internal personal factors but also influenced by the external environment [15]. To obtain more comprehensive information, this paper mainly investigates the influencing factors of unintentional injuries among children and adolescents from three aspects: personal factors, family environment and school environment. Individual characteristics, family and school environment have been reported to be related to the types, causes and patterns of unintentional injury [16]. Previous studies on risk factors for adolescents’ unintentional injury have mainly focused on social demographic variables and some social psychological factors, such as sex, grade, only child, anxiety, depression and mood disorders [14, 16, 17]. Unintentional injury perception also plays a critical role in prevention, with research showing that lack of injury perception is a significant factor in failure to adopt safe practices [18]. The richer the overall understanding of unintentional injury knowledge, the more standardized individual behavior will be, and the lower the incidence of injury events will be [19]. A retrospective cohort study showed that mental health was an independent risk factor for unintentional and recurrent unintentional injuries [20, 21]. A bad mental state may increase the risk of minor injuries and increase the risk of serious injuries [22]. In addition, the family environment also plays a very important role. Research on risk factors for nociceptive behavior in hospitalized patients indicated that negative life events were risk factors for injury [23]. Negative life events easily increase the risk of unintentional injury in children and adolescents [24]. Moreover, classes are places where groups of children gather and play a key role in the growth of children and adolescents. Therefore, the classroom environment might have an important influence on the unintentional injury of children and adolescents. Research has shown that the number of unintentional injuries in schools accounts for more than 30% of total injuries [25]. Some studies have also found a link between school bullying and unintentional injury, which may increase the incidence of injury in children and adolescents [26]. However, few studies have comprehensively understood the influencing factors of unintentional injury among Chinese children and adolescents, especially in LBC. Therefore, it is necessary to generally explore the influencing factors of unintentional injuries to provide a theoretical basis for relevant departments to develop effective and feasible interventions and ultimately reduce the occurrence of unintentional injuries among children and adolescents and LBC [18].

Therefore, the purpose of this study was (1) to evaluate the incidence of unintentional injuries among children and adolescents and the occurrence of various types of unintentional injuries; (2) to comprehensively explore the factors influencing the occurrence of unintentional injuries; and (3) to compare the incidence of unintentional injuries and influencing factors of unintentional injury between LBC and NLBC.

## Materials and methods

### Study design and study sample

This cross-sectional study was conducted in the city of Shenyang in Liaoning Province, Northeast China, from November to December 2019. By stratified random sampling, three elementary schools and three junior high schools were randomly selected from all public day schools in the survey site, which were guaranteed to include key and ordinary schools. To ensure the recovery and accuracy of the questionnaire, we selected students from Grade 4 to Grade 6 in elementary school and Grade 7 to Grade 9 in junior high school. The average of 4 classes were randomly selected in each grade, and all the students were chosen in each selected class. Finally, 3,300 students participated in the study. All participants and their parents were thoroughly informed about the content and aims of this study. The questionnaire was completed by the children (vs. parents), and the surveys were completed anonymously. After obtaining the consent of the respondent, a questionnaire including the International Classification of Disease Version 10 (ICD-10), Unintentional Injury Perception Questionnaire, Multidimensional Subhealth Questionnaire of Adolescent (MSQA), Negative life events, “My Class” questionnaire, and Olweus Bullying/Victim Questionnaire were distributed to these 3300 participants. All the questionnaires are suitable for the age group of our research subjects. In addition, to ensure quality control, at least one investigator in each class was assigned to answer questions at any time during the survey to ensure that each child understands the questions. A total of 514 students were excluded due to problems such as incomplete questionnaires, missing data, and incorrect data registration. There were no demographic differences between invalid respondents and final respondents. The final study subjects consisted of 2,786 Chinese adolescents attending school (1,453 boys and 1,333 girls). The effective response rate was 84.4%. In our population, the average age was 13.19 years old (SD = 1.39, range 10–19 years old). LBC refers to children under the age of 18 years old, and one or both parents have left home to work for at least 6 months. There are generally two situations for left-behind children in China: one is when both parents leave the family and the child is taken care of by grandparents or other relatives; the other is when one parent leaves and the other takes care of them. Our study population included 621 (22.3%) LBC, and 2165 (77.7%) NLBC.

### Study instruments

#### Unintentional injury investigation

The International Classification of Disease Version 10 (ICD-10) classifies unintentional injuries according to the external causes of diseases and deaths (V01-Y98). The classification codes of unintentional injuries coded between V01-X59 are mainly traffic injuries (V01-V99), falling injuries (W00-W19), drowning (W65-W74), accidental suffocation (W75-W84), burn and scalds (X00-X19), animal bites (W53-W55, X20-X29), crushing injury (W23), electric shock (W85-W99), poisoning (X40-X49) and other unintentional injuries [27]. These were also among the fundamental subsets of unintentional injury recommended in the Chinese CDC-National Injury Surveillance System (NISS), Work Manual of National Injury Surveillance System. According to the above criteria and the characteristics of children and adolescents, the 13 most commonly used types of injuries were investigated, including traffic injury, fall injury, drowning, crush injury, poisoning, burn and scalds, cutting injury, animal bites, sprain, accidental suffocation, electric shock, consumer product injuries, and medical malpractice [28]. Children were asked questions such as “I was hit by a motor vehicle” and “I had a bad burn and scalds”. For the convenience of analysis and research, the 4 types of injuries with a small number of occurrences were aggregated as “other types”. Each item has two response categories: (0) no and (1) yes. The abovementioned injury occurred in the past year, and one of the following situations was judged to be the statistical object of the injury, selecting “Yes” as 1 point: (1) to the medical unit for diagnosis and treatment of a certain type of injury; (2) emergency treatment of the injured by family members, teachers, colleagues or peers or nursing; and (3) take sick leave due to injury (work, school, or rest) for more than 0.5 days. Finally, each type of unintentional injury experienced is summed to obtain the total score, which ranges from 0 to 13 points. A single injury was defined as one injury during a year, and multiple injury was defined as ≥ 2 injuries during a year [29, 30]. The measurement of unintentional injury has been widely used among children and adolescents in China [16].

### Unintentional injury perception

The “Unintentional Injury Perception Questionnaire” was compiled by Xu Lingzhong of Shandong University. It includes 9 items with the following contents:“Heard of accidental injuries”, “Thinking about the possibility of unintentional injury while playing”, “Thinking of the possibility of an accident during sports external injury”, “Do some preparation before exercise”, “Unintentional injury may occur after being bitten by pets such as cats and dogs”, “Playing with plastic bullets can hurt people”, “Severe accidental injury causes disability”, “Cross the street occasionally without crossing the zebra crossing”, and “Finger bleeding can be washed with water after paper or cloth bandage” [31]. The higher the score, the lower the unintentional injury cognitive ability. The questionnaire has been widely used among children and adolescents in China [31]. In this study, the Cronbach’s alpha = 0.742.

### Mental subhealth

The Multidimensional Subhealth Questionnaire of Adolescents (MSQA) was developed by Professor Tao Fangbiao of Anhui Medical University and his research group and consists of two parts: physical subhealth and psychological subhealth [32] This study chose the mental health part to assess mental subhealth among adolescents, including emotional problems, conduct problems and social adaptation difficulties, with a total of 39 items. Each item uses Likert’s 6-point scoring. The higher the score, the higher the level of subhealth symptoms. The questionnaire has been widely used among children and adolescents in China [33], and has a high internal consistency in this study(Cronbach’s alpha = 0.966).

### Negative life events

Negative life events include affirmative responses to any of the following incidents that occurred in the respondent’s household in the past year: serious medical problems of participants, suicidal/violent/criminal behavior of family members (e.g., Did any family members commit suicide/violence/crime in the past year?), death of parents, death of other relatives (e.g., Have any other relatives died in the past year?), crowded housing, financial problems, theft, accident/disaster, and departure from parents [34]. If the interviewee gives an affirmative answer to each question in the questionnaire, each question will be given 1 point, and the final score will be 13 points. The higher the score, the more negative life events the interviewee experienced. Negative life events are divided into “0”, “1” and ≥ “2” based on the number of events experienced. Questionnaires have been widely used among students in China. Previous studies have shown that students can understand the content of the questions well [35].

### Classroom environment

The classroom environment was measured by the “My Class” questionnaire developed by Professor Jiang Guangrong [36]. This questionnaire has frequently been used to measure the social psychological classroom environment of Chinese school students. There are 38 items in the questionnaire, using Likert’s 5-point scoring method. It includes five factors: (1) teacher-student relationship, (2) peer relationship, (3) discipline and order, (4) competition, and (5) learning burden. High scores represent a high level of that dimension. The questionnaire has been widely used among children and adolescents in China[36], and has a high internal consistency in this study. I The Cronbach’s alpha = 0.872.

### Bullying

Bullying behavior was investigated using the revised Olweus Bullying/Victim Questionnaire [37]. There are 7 items in the scale, and the total score ranges from 0 to 42, with higher scores indicating more severe experiences of being bullied. We applied the cutoff score of the median to define the “bullying victim” group [38]. The questionnaire has been widely used among children and adolescents in China [39]. In this study, the Cronbach’s alpha = 0.804.

### Demographic and other personal factors

Child factors included gender, school level (elementary/junior high school), only child(yes/no), and LBC (yes/no). Socioeconomic status (SES) was determined by the parents’ highest education level and was divided into low, middle, and high SES [40].

### Measure

#### Statistical analysis

The χ^2^ test was used to test the distribution differences in the types of unintentional injuries between LBC and NLBC. There were three types of unintentional injury outcomes: 0 (no injury), 1 (single injury), and ≥ 2 (multiple injuries). We used the χ^2^ test to discuss distributions of the three accidental injuries in each factor. All variables related to unintentional injuries in univariate analysis (P < 0.25) were entered into the multivariate model. Due to the need for the multivariate logistic regression model, all continuous variables were divided into high and low levels according to the median. Due to the small number of LBC, the accuracy and credibility of the results of multiple injuries would be affected due to the insufficient sample size.Therefore, binary logistic regression was used to analyze the influencing factors of unintentional injuries among LBC and NLBC. Odds ratios (ORs) and 95% confidence intervals (95% CIs) were calculated to estimate the associations between relevant risk factors and unintentional injury. All statistical analyses were performed using SPSS software (version 26.0) (IBM SPSS, Inc. Chicago, IL, USA), two-sided tests, and *P* ≤ 0.05 was considered statistically significant.

## Results

**Fig. 1:**
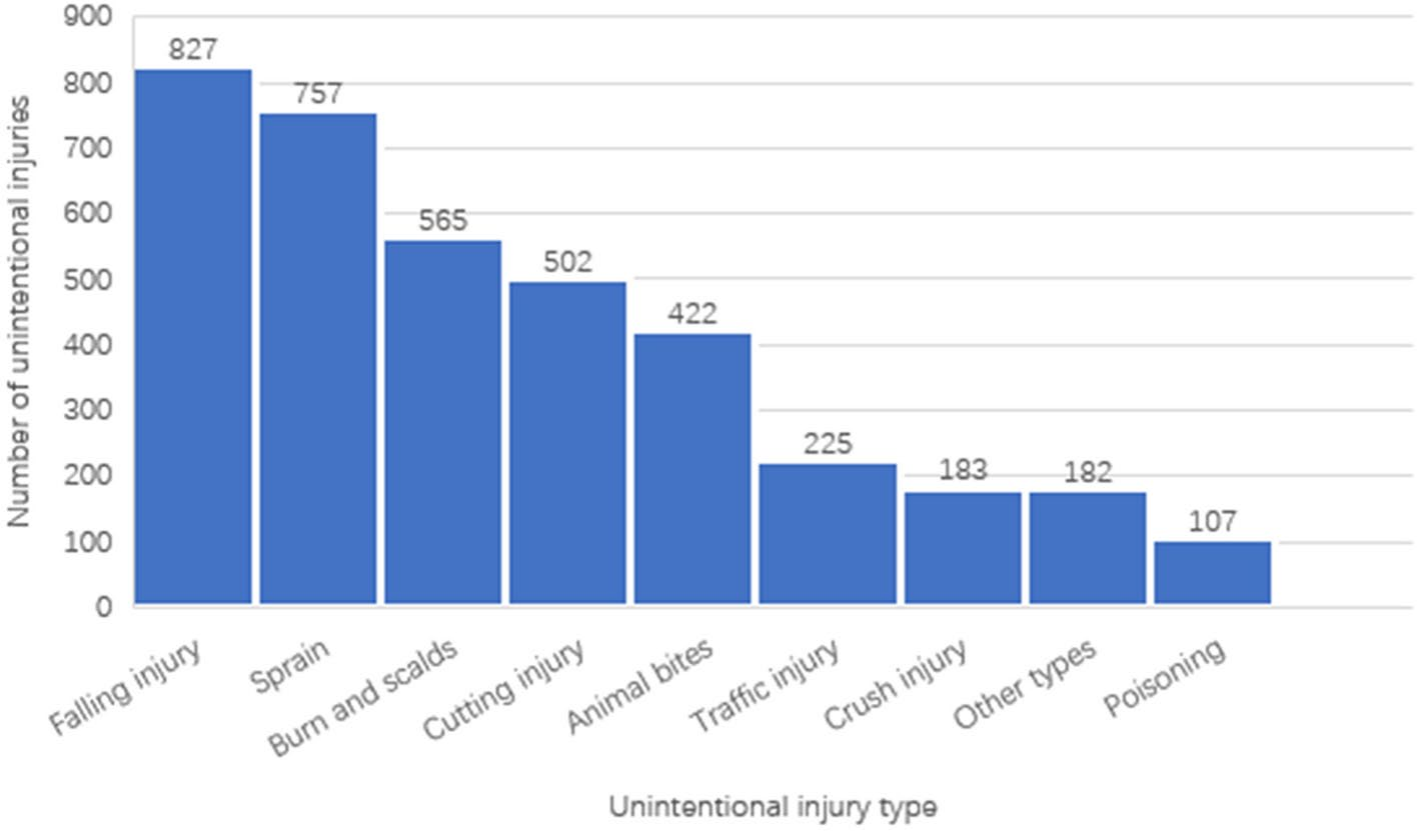
Number of unintentional injury types

Of the population surveyed, 1,738 (62.4%) participants suffered at least one type of unintentional injury. Falls (827, 29.7%) and sprains (757, 27.2%) were the most common injuries reported, followed by burns and scalds (565, 20.3%). These details are shown in Fig. 1.

The overall incidence of unintentional injuries among LBC was higher than that among NLBC (*p* < 0.05), and the incidences of burns and scalds, cutting injuries and animal bites among LBC were higher than those among NLBC (*p* < 0.05). These details are shown in Table 1.

**Table 1:** Comparison of unintentional injuries between LBC and NLBC

Unintentional Injury	N (%)	LBC N (%)	NLBC N (%)	*p*
**All**	1738(62.4)	419(67.5)	1319(60.9)	0.003
Traffic injury	225(8.1)	57(9.2)	168(7.8)	0.253
Falling injury	827(29.7)	197(31.7)	630(29.1)	0.207
Poisoning	107(3.8)	32(5.2)	75(3.5)	0.054
Burn and scalds	565(20.3)	162(26.1)	403(18.6)	0.000
Crush injury	183(6.6)	45(7.2)	138(6.4)	0.439
Cutting injury	502(18.0)	133(21.4)	369(17.0)	0.012
Animal bites	422(15.1)	120(19.3)	302(13.9)	0.001
Sprain	757(27.2)	165(26.6)	592(27.3)	0.702
Other types	182(6.5)	63(10.1)	119(5.5)	0.000

Abbreviation: *LBC* Left-behind children. *NLBC*, Not-left-behind children;

Other types: Drowning, Accidental suffocation, Electric shock, Consumer product injuries, Medical malpractice

Table 2 shows the distribution differences of unintentional injuries in risk factors. The incidence of unintentional injuries among girls was higher than that among boys. (*p* < 0.05). The incidence of multiple unintentional injuries among LBC was higher than that among NLBC (*p* < 0.05). The incidence of unintentional injury incidents in junior high school was significantly higher than that in elementary school (*p* < 0.05). In addition, mental subhealth, negative life events and bullying events were positively related to the occurrence of unintentional injuries; that is, a higher levels of mental health symptoms, more negative life events and bullying events were all related to a high incidence of unintentional injuries. Discipline and order were negatively related to the occurrence of unintentional injuries, and low-level discipline and order were related to a high incidence of unintentional injuries. Table 3 shows the results of multivariate logistic regression to identify factors that may influence unintentional injury. Low level of unintentional injury perception (OR = 1.234), Junior high school (OR = 1.237), higher levels of mental health symptoms (OR = 1.247), bullying events (OR = 1.414) and repeated negative life events (OR = 1.661) were related to the occurrence of an unintentional injury event. Girl (OR = 1.252), low level of discipline and order (OR = 1.277), junior high school (OR = 1.296), low level of unintentional injury perception (OR = 1.321), higher levels of mental health symptoms (OR = 1.442), experience of bullying (OR = 2.340) and multiple negative life events (OR = 2.724) were related to the occurrence of multiple unintentional injuries.

**Table 2:** Univariate analysis of unintentional injuries among children and adolescents (N = 2786)

	**Variable**	**Damage(%)**			**χ**^**2**^	***p***
		**0**	**1**	** ≥ 2**		
**Individual factors**	**School Level**					
	Elementary school Junior high school	477(40.5) 571(35.5)	288(24.4) 410(25.5)	413(35.1) 627(39.0)	7.603	0.022
	**Sex**					
	Boy Girl	584(40.2) 464(34.8)	359(24.7) 339(25.4)	510(35.1) 530(39.8)	9.547	0.008
	**The one-child**					
	Yes No	614(37.9) 434(37.2)	424(26.2) 274(23.5)	581(35.9) 459(39.3)	4.242	0.120
	**LBC**					
	Yes No	202(32.5) 846(39.1)	153(24.6) 545(25.2)	266(42.8) 774(35.8)	12.042	0.002
	**Unintentional injury perception**					
	High	545(41.0)	321(24.2)	462(34.8)	13.077	0.001
	Low	503(34.5)	377(25.9)	578(39.6)		
	**Subhealth**					
	High	456(32.3)	352(24.9)	605(42.8)	44.924	0.000
	Low	592(43.1)	346(25.2)	435(31.7)		
**Family environment**	**Household type**					
	Small family	724(37.5)	468(24.3)	737(38.2)	4.555	0.336
	Big family	225(38.5)	161(27.6)	198(33.9)		
	other	99(36.3)	69(25.3)	105(38.5)		
	**SES**					
	low	455(38.2)	290(24.4)	445(37.4)	2.324	0.676
	Middle	455(37.4)	301(24.8)	459(37.8)		
	High	138(36.2)	107(28.1)	136(35.7)		
	**Number of negative life events**					
	0	299(51.0)	146(24.9)	141(24.1)	104.264	0.000
	1	378(40.7)	224(24.1)	326(35.1)		
	≥ 2	371(29.2)	328(25.8)	573(45.0)		
**School environment**	**Teacher-student relationship**					
	High	468(35.3)	352(26.5)	506(38.2)	6.344	0.042
	Low	580(39.7)	346(23.7)	534(36.6)		
	**Peer relationship**					
	High	476(37.3)	338(26.5)	461(36.2)	2.905	0.234
	Low	572(37.9)	360(23.8)	579(38.3)		
	**Discipline and Order**					
	High	524(39.7)	355(26.9)	441(33.4)	16.605	0.000
	Low	524(35.7)	343(23.4)	599(40.9)		
	**Competition**					
	High	544(39.1)	333(23.9)	516(37.0)	3.055	0.217
	Low	504(36.2)	365(26.2)	524(37.6)		
	**Learning Burden**					
	High	477(36.4)	318(24.3)	516(39.4)	4.361	0.113
	Low	571(38.7)	380(25.8)	524(35.5)		
	**Bullying**					
	Yes	453(30.5)	357(24.1)	674(45.4)	99.357	0.000
	No	595(45.7)	341(26.2)	366(28.1)		

Abbreviation: *SES* Socioeconomic status. other, single parent foster homes and others

**Table 3:** Analysis of the influencing factors of unintentional injuries among children and adolescents (*N* = 2786)

**Number of injuries**		**Variable**	***P***	**OR (95% CI)**
**1**	**Individual factors**	**School Level**		
		Elementary school	1	1
		Junior high school	0.05	1.237(1.000–1.531)
		**Unintentional injury perception**		
		Low	0.037	1.234(1.013–1.505)
		High	1	1
		**Subhealth**		
		Low	1	1
		High	0.035	1.247(1.015–1.531)
	**Family environment**	**Number of negative life events**		
		≥ 2	0.000	1.661(1.286–2.147)
		1	0.199	1.186(0.914–1.539)
		0	1	1
	**School environment**	**Bullying**		
		Yes	0.001	1.414(1.147–1.743)
		No	1	1
** ≥ 2**	**Individual factors**	**School Level**		
		Elementary school	1	1
		Junior high school	0.009	1.296(1.066–1.574)
		**Sex**		
		Boy	1	1
		Girl	0.016	1.252(1.042–1.504)
		**Unintentional injury perception**		
		Low	0.003	1.321(1.013–1.586)
		High	1	1
		**Subhealth**		
		Low	1	1
		High	0.000	1.442(1.193–1.744)
	**Family environment**	**Number of negative life events**		
		≥ 2	0.000	2.724(2.121–3.499)
		1	0.000	1.739(1.346–2.246)
		0	1	1
	**School environment**	**Discipline and Order**		
		Low	0.022	1.277(1.036–1.574)
		High	1	1
		**Bullying**		
		Yes	0.000	2.340(1.925–2.845)
		No	1	1

Age and gender were controlled as confounding factors

The univariate analysis results for LBC and NLBC show that unintentional injury perception, subhealth, negative life events, discipline and order, and bullying in LBC were significantly related to unintentional injury. School level, sex, unintentional injury perception, subhealth, negative life events, teacher-student relationship, discipline and order and bullying in NLBC were significantly related to unintentional injury.

The results of binary logistic regression comparing LBC with NLBC are shown in Table 4. In LBC, a low level of unintentional injury perception (OR = 1.439), multiple experiences of negative life events (OR = 2.200) and bullying (OR = 2.337) were associated with the occurrence of unintentional injury events. In NLBC, girls (OR = 1.202), low-level unintentional injury perception (OR = 1.252), junior high school (OR = 1.294), higher levels of mental health symptoms (OR = 1.347), bullying (OR = 1.780) and negative life events (OR = 2.150) were related to unintentional injury events.

**Table 4:** Comparison of Influencing Factors of Unintentional Injury between LBC (*N* = 621) and NLBC (*N* = 2165)

**Left behind children**		**Variable**	***P***	**OR (95% CI)**
**Yes**	**Individual factors**	**Unintentional injury perception**		
		Low	0.031	1.439(1.037–2.150)
		High	1	1
	**Family environment**	**Number of negative life events**		
		≥ 2	0.000	2.200(1.218–3.975)
		1	0.473	1.238(0.691–2.218)
		0	1	1
	**School environment**	**Bullying**		
		Yes	0.000	2.337(1.582–3.452)
		No	1	1
**No**	**Individual factors**	**School Level**		
		Elementary school	1	1
		Junior high school	0.008	1.294(1.068–1.567)
		**Sex**		
		Boy	1	1
		Girl	0.048	1.202(1.002–1.442)
		**Unintentional injury perception**		
		Low	0.016	1.252(1.042–1.503)
		High	1	1
		**Subhealth**		
		Low	1	1
		High	0.002	1.347(1.115–1.628)
	**Family environment**	**Number of negative life events**		
		≥ 2	0.000	2.150(1.709–2.704)
		1	0.001	1.483(1.179–1.865)
		0	1	1
	**School environment**	**Bullying**		
		Yes	0.000	1.780(1.469–2.156)
		No	1	1

Age and gender were controlled as confounding factors

## Discussion

Unintentional injury has gradually become a very important public health problem in China. It is estimated that by 2050, the number of deaths due to injuries in China will reach 2.5 million each year [41]. This study estimated the status of unintentional injury among Chinese children and adolescents and explored the associated factors from three aspects: personal factors, family and school environment. Due to the vast territory, diverse climate and numerous nationalities in China, the incidence of unintentional injuries among children and adolescents varies greatly in different regions. Our study showed that the incidence of unintentional injuries was 62.4%, Compared with domestic studies in China, it is consistent with reports in Chengdu (62.8%) [42] and Hunan (60.1%) [43], lower than that in Guangxi Zhuang Autonomous Region (67.67%) [44]and higher than that in Suzhou (9.64%) [45], Guangdong Province (26.24%) [46] and Nantong City (45.75%) [47]. Meanwhile, compared with foreign research, the incidence was lower than that in African countries such as Liberia (71.1%) [29] and Egypt (68.5%) [48] but higher than that in South American countries such as Argentina (27.1%) and Bolivia (36.8%) [49] and in Asian countries such as Malaysia (34.9%) [50] and Sri Lanka (35.6%) [6]. Consistent with previous studies [29, 51], falls and burns were the most common accidental injuries in our survey. The occurrence of fall injury is not limited by time, place and environment, and it occurs frequently because it can be caused by many reasons [52]. Compared with other studies [6], the incidence of traffic injuries in this study was relatively lower, and the incidence of sprains was higher. This may be related to various factors, such as different geographical environments, topography, social and economic development levels, convenient transportation and lifestyles [17, 29]. Moreover, perhaps some previous studies failed to follow the definition and classification of unintentional injury in the ICD-10. In addition, collecting information from multiple sources, such as adolescents, teachers and parents might make the results different.

Our study also compared the difference between LBC and NLBC. Previous research results showed that the incidence of unintentional injuries among LBC was significantly higher than that among NLBC, and the number of left-behind children who suffered unintentional injuries many times was significantly higher than that of non left-behind children [53]. This study found that LBC were more likely to suffer unintentional injury than NLBC and the incidence of burn and scalds, cutting injury and animal bites was higher in LBC. The results of logistic analysis also found that LBC were more likely to be exposed to risk environments, which might cause more unintentional injury. In terms of personal factors, compared with NLBC, LBC have a lower cognitive level of unintentional injury, fewer opportunities to receive safety education, and weak safety awareness [19]. Insufficient life experience, a limited ability to identify risks, strong curiosity, and vulnerability to a variety of temptations and attractions in LBC may increase their risk of unintentional injury [54]. In terms of family environment, studies have shown that children exposed to negative life events were at higher risk of injury, while LBC were more likely to be exposed to negative life events [55]. Children who have experienced negative life events are often eccentric, unable to concentrate on their studies, and even have various behavioral disorders [56]. Compared with NLBC, LBC may have experienced a serious medical accident, and their family members committed suicide/violence/crime, separated themselves from their parents and other negative experiences, and even faced high-level negative events from interpersonal conflict, academic pressure, loss, and health problems [55]. There was a positive correlation between negative life events and the incidence of unintentional injury risk behaviors, which might suggest that the more negative life events experienced, the greater the likelihood of unintentional injury [57]. But because negative life events were composed of a series of experiences, there may be more complex path associations between variables. Future research can further explore the underlying mechanisms. In terms of the school environment, compared with adolescents who did not experience bullying, adolescents who have been bullied are more likely to suffer serious harm [58]. A survey found that more than one-third of LBC reported experiences of bullying, and LBC were more vulnerable to bullying [59, 60]. Victims of bullying were associated with a predisposition to physical conflict, which may lead to the occurrence of unintentional injuries [61]. Therefore, we should pay more attention to LBC, improve their awareness of unintentional injuries, and take effective measures to reduce the occurrence of negative life events and bullying to prevent the occurrence of accidental injuries.

Consistent with previous studies [62, 63], individual factors affect behavior, and research found that older adolescents were more vulnerable to unintentional injuries. Children’s cognitive abilities, range of activities and behaviors all change dramatically with age, and their growing mobility and curiosity cannot keep up with their recognition of danger or the knowledge reserve of injury, which is prone to fall into danger [64]. In addition, our research also found that the overall incidence of unintentional injuries and the incidence of cuts and sprains were higher in girls than in boys. Although some previous studies showed that boys were more likely to suffer unintentional injuries than girls [27, 65, 66], other studies also found that girls had a higher incidence of unintentional injuries than boys, especially burn and scalds [67]. This may be because boys are more mischievous in our traditional view, so parents pay more attention to the supervision and safety education of boys. Richard Rowe et al. [68] found that children with mental disorders had a higher incidence of unintentional injuries. Destructive and emotional psychological problems increase the risk of unintentional injury [69]. In China, Chen G et al. [70] found that psychological symptoms were associated with an increased risk of nonfatal unintentional injury. Other studies have also indicated that the baseline psychological subhealth state has a predictive effect on adolescent unintentional injury [71]. In addition, the external environment also affects the occurrence of behavior. China is generally a collectivist culture, where individual rights are subordinate to collective rights. Collectivism emphasizes interdependence and overall interests. Therefore, Chinese culture tends to value the important role of discipline and order in the development of children and adolescents, and the lack of strict discipline and order may have a strong impact on students’ behavior and mental health [58]. Several previous studies has identified a link between the discipline and order and incidents of unintentional injury [72]. Several previous studies have identified a link between discipline and order and incidents of unintentional injury [73]. The risk of unintentional injury of students in a good class environment was significantly lower than that of students in a poor class environment [74]. Previous studies thought that interpersonal relationships in the class environment play a very important role, but this study did not find any effect, and subsequent research needs to be further explored. Therefore, in the planning of injury prevention interventions, we should pay attention to the recognition of children and adolescents’ mental health interventions, improve the safety education and supervision of high-grade students and girls, and form a good class atmosphere.

This study has several limitations. First, this study adopted a cross-sectional design, and it could not draw a causal relationship between the survey factors and the outcome measurement. Whether some factors, such as mental subhealth, were risk factors for unintentional injury or the consequence could not be determined. Moreover, it was not possible to determine whether the effects of each factor on adolescents over time still exist. Our study is only a preliminary exploration of influencing factors to provide clues for further mechanism research. Therefore, next longitudinal studies should be conducted to further explore the interaction path and verify the causality between these variables. Second, this study used a self-report questionnaire to collect variable information, and the results might deviate from the true situation. Therefore, data obtained from parents and teachers will be an important direction in future research. Finally, some important information was not collected, such as which parent migrated and who was the caregiver during parent migration. According to the definition of LBC, regardless of whether both parents or one left for more than 6 months, they are counted as LBC. The purpose of this study was to obtain an initial understanding of the unintentional injury status of LBC. Future research will collect these information for further in-depth analysis. Despite these limitations, based on the fairly large and representative sample of this study, this study can offer some important information for research on unintentional injuries in Chinese children and adolescents.

## Conclusions

This study found that there was a high incidence of unintentional injury among school children and adolescents. Personal characteristics, family environment and school environment were closely associated with the occurrence of injury. Particular attention should be given to LBC injuries, and targeted measures should be encouraged to provide LBC with necessary emotional and social support. It is recommended that families and schools should improve the self-protection awareness of children and adolescents, carry out safety knowledge education and related theme activities to prevent bullying behaviors and the occurrence of negative life events for the purpose of creating a positive atmosphere and providing more care and help to students, especially LBC.

## Data Availability

The data are not publicly available because they contain information that could compromise research participant privacy and consent, but are available from the corresponding author upon reasonable request.

## References

[1] Alaghehbandan R (2010). Unintentional injuries among children and adolescents in Aboriginal and non-Aboriginal communities, Newfoundland and Labrador. Canada Int J Circumpolar Health.

[2] Zhang L, et al. Prevalence and influencing factors of unintentional injury among primary school students in rural areas of Yunnan province. Applied preventive medicine. 2020;26(03):177–179+184.

[3] Jiang X (2010). An analysis of 6215 hospitalized unintentional injuries among children aged 0–14 in northwest China. Accid Anal Prev.

[4] de Ramirez SS (2012). Unintentional injuries: magnitude, prevention, and control. Annu Rev Public Health.

[5] Salam RA (2016). Interventions to Prevent Unintentional Injuries Among Adolescents: A Systematic Review and Meta-Analysis. J Adolesc Health.

[6] Wickramasinghe S (2020). Serious non-fatal unintentional injuries among in-school adolescents in Sri Lanka: results from the 2016 Sri Lankan global school-based health survey. BMC Public Health.

[7] Gore FM (2011). Global burden of disease in young people aged 10–24 years: a systematic analysis. Lancet.

[8] Xu R, et al. Study on Disease Burden of Adolescents in China in 2015. Chin J Prev Med. 2017;51(10):910-14. 10.3760/cma.j.issn.0253-9624.2017.10.008 29036993

[9] Leilei D (2019). The burden of injury in China, 1990–2017: findings from the Global Burden of Disease Study 2017. Lancet Public Health.

[10] Zhang M (2019). Unintentional injuries: A profile of hospitalization and risk factors for in-hospital mortality in Beijing. China Injury.

[11] He L (2021). Clustering of multiple lifestyle behaviors among migrant, left-behind and local adolescents in China: a cross-sectional study. BMC Public Health.

[12] Yang R. The current status and influential factors of unintentional injuries of left-behind preschool children in city of Jilin province. Master's thesis. Jilin University; 2005.

[13] Juan J (2021). Prevalence of unintentional injury among left-behind children in mainland China: Evidence from epidemiological surveys. Child Care Health Dev.

[14] Ma S, et al. Left-Behind Children and Risk of Unintentional Injury in Rural China-A Cross-Sectional Survey. Int J Environ Res Public Health. 2019;16(3):403. 10.3390/ijerph16030403 PMC6388167 30708979

[15] Bandura A (1989). Human agency in social cognitive theory. Am Psychol.

[16] Hu H (2018). A comparative study of unintentional injuries among schooling left-behind, migrant and residential children in China. Int J Equity Health.

[17] Aboagye RG, et al. Prevalence and Correlates of Unintentional Injuries among In-School Adolescents in Ghana. Int J Environ Res Public Health. 2021;18(13):6800. 10.3390/ijerph18136800 PMC8297100 34202752

[18] McKenzie LB (2019). Maternal Knowledge, Attitudes, and Behavioral Intention after Exposure to Injury Prevention Recommendations in the News Media. J Health Commun.

[19] Yang X (2018). Analysis on the cognitive status and influencing factors of unintentional injury among primary and secondary school students in Yunnan Province. Chinese Primary Health Care.

[20] Wan JJ (2006). Mental illness as an independent risk factor for unintentional injury and injury recidivism. J Trauma.

[21] Huang Z (2010). Analysis on psychological susceptibility of adolescents to injury related factors in Shaoxing City. School Health in China.

[22] Tao S (2016). Interactions of problematic mobile phone use and psychopathological symptoms with unintentional injuries: a school-based sample of Chinese adolescents. BMC Public Health.

[23] Liu RT (2014). Negative life events and non-suicidal self-injury in an adolescent inpatient sample. Arch Suicide Res.

[24] Gu D (2020). Research Progress on the Influencing Factors and Intervention Strategies of Accident-prone Children’s Accidental Injuries. Hainan Medical Journal.

[25] Zhang D (2007). Epidemiological survey of accidental injury among preschool children. Chinese Journal of Social Medicine.

[26] Peltzer K, Pengpid S. Nonfatal Injuries and Psychosocial Correlates among Middle School Students in Cambodia and Vietnam. Int J Environ Res Public Health. 2017;14(3):280. 10.3390/ijerph14030280 PMC5369116 28282872

[27] Borse NN (2009). Unintentional childhood injuries in the United States: key findings from the CDC childhood injury report. J Safety Res.

[28] Dessypris N (2009). Combating unintentional injury in the United States: lessons learned from the ICD-10 classification period. J Trauma.

[29] Pengpid S, Hinneh JT, Peltzer K (2021). Prevalence and correlates of single and multiple unintentional non-fatal injuries among school-going adolescents in Liberia. Injury.

[30] Fei G (2022). Unintentional injuries and risk behaviours of internal migrant children in southern China: A cross-sectional study. Health Soc Care Community.

[31] Ma S. Investigation and research on accidental injury status and injury cognition of primary and middle school students in Baotou City. Master's thesis. Shandong University; 2008.

[32] Tao F (2008). The Compilation and Application of Sub-health Multidimensional Evaluation Questionnaire of Chinese Adolescents Chinese Journal of Disease. Control.

[33] Wang H (2011). Application and evaluation of multi - dimensional sub-health evaluation questionnaire for adolescent middle school students in reservoir area. Chinese General Practice.

[34] Wang JN, Liu L, Wang L (2014). Prevalence and associated factors of emotional and behavioural problems in Chinese school adolescents: a cross-sectional survey. Child Care Health Dev.

[35] Wang J (2014). Agreement between parents and adolescents on emotional and behavioral problems and its associated factors among Chinese school adolescents: a cross-sectional study. BMC Psychiatry.

[36] Jiang G (2004). Classroom environment in primary and secondary schools: Structure and measurement. Psychol Sci.

[37] Zhang W, Wu J. Revision of the Chinese version of Olweus Child Bullying Questionnaire. Psychol Dev Educ. 1999;(2):8-1238.

[38] Hanmei T (2018). Relationship between bullying and suiciderelated behaviors among middle school students. Chin J School Health.

[39] Tang H (2018). Association between school bullying and suicide-related behaviors among middle school students in Jiangxi Province. Chin J School Health.

[40] Weine AM, Phillips JS, Achenbach TM (1995). Behavioral and emotional problems among Chinese and American children: parent and teacher reports for ages 6 to 13. J Abnorm Child Psychol.

[41] Liu Q (2012). The gap in injury mortality rates between urban and rural residents of Hubei Province. China BMC Public Health.

[42] Zhao X (2016). Investigation on current situation of children accidental injury in Chengdu. Nurs Res.

[43] Yang X. Study on Status and Influence of Accident Injury in School-age Children in Hunan Rural. Master's thesis. Hunan Normal University; 2014.

[44] Guo G, Wei D (2017). Clinical analysis of 2286 cases of accidental injuries in children. Chinese Pediatric Emergency Medicine.

[45] Gong T (2020). Epidemiological survey of accidental injury in children aged 0–14 years in Suzhou. Chinese Journal of Social Medicine.

[46] Zeng C (2022). Epidemiological characteristics of inpatients with accidental injuries in children in Guangdong Maternal and Child Health Hospital from 2015 to 2020. China Modern Medicine.

[47] Mao J (2018). Effect evaluation of intervention for children accidental injury in Nantong City. Chinese Journal of Health Education.

[48] Wahdan MM (2016). Prevalence of injuries among high school students in Eastern and Western parts of Cairo. Egypt Injury.

[49] Beck NI (2016). Adolescent injuries in Argentina, Bolivia, Chile, and Uruguay: Results from the 2012–2013 Global School-based Student Health Survey (GSHS). Injury.

[50] Peltzer K, Pengpid S (2015). Unintentional Injuries and Psychosocial Correlates among in-School Adolescents in Malaysia. Int J Environ Res Public Health.

[51] Pengpid S, Peltzer K (2020). High prevalence of unintentional injuries and socio-psychological correlates among school-going adolescents in Timor-Leste. Int J Adolesc Med Health.

[52] Lin L, Lin G, Ni L (2006). Analysis of injuries and related risk behaviors among adolescents in Guangzhou School health in China. Chin J School Health.

[53] Fellmeth G (2018). Health impacts of parental migration on left-behind children and adolescents: a systematic review and meta-analysis. Lancet.

[54] Zhang F (2021). Study on risk Prevention mechanism of accidental injury of rural Left-behind children – A case study of K Province. J Soc Work.

[55] Guang Y (2017). Depressive symptoms and negative life events: What psycho-social factors protect or harm left-behind children in China?. BMC Psychiatry.

[56] Zhang H (2016). Unintentional childhood injury: a controlled comparison of behavioral characteristics. BMC Pediatr.

[57] Wang S, et al. Analysis of The Situation of Health-risk Behaviors and Its Relationship with Negative Life Events among the Students in Specialty Medical Science College of Yunnan. Journal of Kunming Medical University. 2016;37(6):43-7.

[58] Muula AS, Siziya S, Rudatsikira E (2011). Prevalence and socio-demographic correlates for serious injury among adolescents participating in the Djibouti 2007 Global School-based Health Survey. BMC Res Notes.

[59] Zhang H, Zhou H, Cao R (2021). Bullying Victimization Among Left-Behind Children in Rural China: Prevalence and Associated Risk Factors. J Interpers Violence..

[60] Chester KL (2015). Cross-national time trends in bullying victimization in 33 countries among children aged 11, 13 and 15 from 2002 to 2010. Eur J Public Health.

[61] Jansen L, Barnighausen T, Lowery Wilson M (2020). Injuries among adolescents in Greenland: behavioural and socio-economic correlates among a nationally representative sample. PeerJ..

[62] Centers for Disease Control and Prevention (CDC). Years of potential life lost from unintentional injuries among persons aged 0-19 years - United States, 2000-2009. MMWR Morb Mortal Wkly Rep. 2012;61(41):830-3. 23076091

[63] Haynes R, Reading R, Gale S (2003). Household and neighbourhood risks for injury to 5–14 year old children. Soc Sci Med.

[64] Wang J (2017). Analysis of injury deaths among children and adolescents in Beijing from 2011 to 2015. Capital Journal of Public Health.

[65] He S (2014). Global childhood unintentional injury study: multisite surveillance data. Am J Public Health.

[66] Saß AC, Poethko-Müller C, Rommel A (2014). Unintentional injuries in childhood and adolescence: current prevalence, determinants, and trends: results of the KiGGS study: first follow-up (KiGGS Wave 1). Bundesgesundheitsblatt Gesundheitsforschung Gesundheitsschutz.

[67] Golshan A, Patel C, Hyder AA (2013). A systematic review of the epidemiology of unintentional burn injuries in South Asia. J Public Health (Oxf).

[68] Rowe R, Maughan B, Goodman R (2004). Childhood psychiatric disorder and unintentional injury: findings from a national cohort study. J Pediatr Psychol.

[69] Rowe R, Simonoff E, Silberg JL (2007). Psychopathology, temperament and unintentional injury: cross-sectional and longitudinal relationships. J Child Psychol Psychiatry.

[70] Chen G (2005). Psychological symptoms and nonfatal unintentional injuries among Chinese adolescents: a prospective study. J Adolesc Health.

[71] Xu S (2012). The predictive effect of mental sub-health on self-injury and accidental injury behavior in adolescents. Chinese Journal of Epidemiology.

[72] Wang J, Hu S, Wang L (2018). Multilevel analysis of personality, family, and classroom influences on emotional and behavioral problems among Chinese adolescent students. PLoS ONE.

[73] Mytton J (2009). Unintentional injuries in school-aged children and adolescents: lessons from a systematic review of cohort studies. Inj Prev.

[74] Ji Y (2016). The Migrant Paradox in Children and the Role of Schools in Reducing Health Disparities: A Cross-Sectional Study of Migrant and Native Children in Beijing, China. PLoS ONE.

